# Structure and Function of RhoBTB1 Required for Substrate Specificity and Cullin-3 Ubiquitination

**DOI:** 10.1093/function/zqad034

**Published:** 2023-07-03

**Authors:** Gaurav Kumar, Shi Fang, Daria Golosova, Ko-Ting Lu, Daniel T Brozoski, Ibrahim Vazirabad, Curt D Sigmund

**Affiliations:** Department of Physiology, Cardiovascular Center, Medical College of Wisconsin, Milwaukee, WI 53226, USA; Department of Physiology, Cardiovascular Center, Medical College of Wisconsin, Milwaukee, WI 53226, USA; Department of Physiology, Cardiovascular Center, Medical College of Wisconsin, Milwaukee, WI 53226, USA; Department of Physiology, Cardiovascular Center, Medical College of Wisconsin, Milwaukee, WI 53226, USA; Department of Physiology, Cardiovascular Center, Medical College of Wisconsin, Milwaukee, WI 53226, USA; Department of Physiology, Cardiovascular Center, Medical College of Wisconsin, Milwaukee, WI 53226, USA; Department of Physiology, Cardiovascular Center, Medical College of Wisconsin, Milwaukee, WI 53226, USA

**Keywords:** protein–protein interaction, Cullin-3, RhoBTB1, co-immunoprecipitation, proximity labeling

## Abstract

We identified Rho-related BTB domain containing 1 (RhoBTB1) as a key regulator of phosphodiesterase 5 (PDE5) activity, and through PDE5, a regulator of vascular tone. We identified the binding interface for PDE5 on RhoBTB1 by truncating full-length RhoBTB1 into its component domains. Co-immunoprecipitation analyses revealed that the C-terminal half of RhoBTB1 containing its two BTB domains and the C-terminal domain (B1B2C) is the minimal region required for PDE5 recruitment and subsequent proteasomal degradation via Cullin-3 (CUL3). The C-terminal domain was essential in recruiting PDE5 as constructs lacking this region could not participate in PDE5 binding or proteasomal degradation. We also identified Pro^353^ and Ser^363^ as key amino acid residues in the B1B2C region involved in CUL3 binding to RhoBTB1. Mutation of either of these residues exhibited impaired CUL3 binding and PDE5 degradation, although the binding to PDE5 was preserved. Finally, we employed ascorbate peroxidase 2 (APEX2) proximity labeling using a B1B2C–APEX2 fusion protein as bait to capture unknown RhoBTB1 binding partners. Among several B1B2C-binding proteins identified and validated, we focused on SET domain containing 2 (SETD2). SETD2 and RhoBTB1 directly interacted, and the level of SETD2 increased in response to pharmacological inhibition of the proteasome or Cullin complex, CUL3 deletion, and RhoBTB1-inhibition with siRNA. This suggests that SETD2 is regulated by the RhoBTB1–CUL3 axis. Future studies will determine whether SETD2 plays a role in cardiovascular function.

## Graphical Abstract

**Graphical Abstract:**
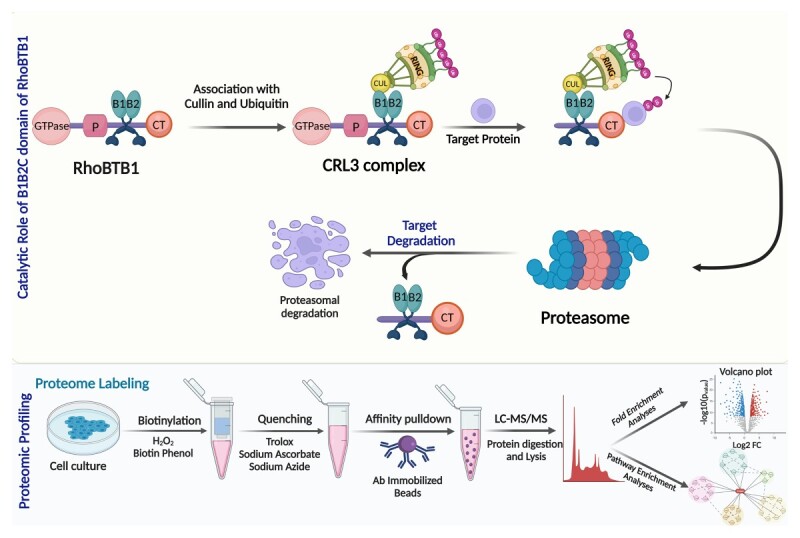

## Introduction

Hypertension (HTN) or high blood pressure (BP) is among the most prevalent factors underlying cardiovascular impairments. As reported by the American Heart Association, HTN is responsible for 69% of first heart attack, 77% of first stroke, and 74% of chronic heart failure.^1^ Data from the Centers for Disease Control and Prevention suggest that nearly half of the US population has HTN and only 24% of hypertensive subjects have their BP under control.^2^ The lifetime risk of heart failure is also twice in hypertensive subjects as compared to normotensive subjects.^3^ In 2019, HTN was the primary or contributing cause of mortality of more than 0.5 million US adults. As averaged over 12 yr from 2003 to 2014, the cost of HTN treatment is about $131 billion annually, which is expected to reach $1.1 trillion by 2035.^4,5^ Consequently, it is essential that continued research identifies new pathways and potential therapeutic targets to manage HTN.

Peroxisome proliferator-activated receptor γ (PPARγ) is a ligand-activated transcription factor, which controls both the bioavailability of nitric oxide and the responsiveness of the vascular smooth muscle to endothelial-derived nitric oxide, and thus plays an important regulator of blood vessel tone.^6^ Previous genetic studies have established the importance of PPARγ in cardiovascular diseases, including HTN.^7–9^ Early studies suggested that PPARγ may mediate cardioprotective effects on the vasculature.^10^ More recently, expression of dominant negative PPARγ isoforms in either endothelium or vascular smooth muscle caused an enhanced susceptibility to, or directly caused, vascular dysfunction and HTN.^11–13^ But, the generalized activation of PPARγ by high affinity pharmacological agonists is associated with adverse cardiovascular outcomes.^14^ The intent of our research is to optimize the advantageous outcomes of PPARγ activation while reducing its unfavorable impacts by focusing on transcriptional targets of PPARγ.

We discovered that Rho-related BTB domain 1 (RhoBTB1) is an important PPARγ target gene in vascular smooth muscle cells.^12^ Induction of RhoBTB1 expression in mice carrying a smooth muscle specific dominant negative mutation in PPARγ reversed HTN, vascular dysfunction, and arterial stiffness.^15^ Inducible expression of RhoBTB1 in a model of pre-existing angiotensin-II-dependent hypertension quickly reversed arterial stiffness.^16^ RhoBTB1 is a broad complex, tramtrack, and bric a brac (BTB)-domain containing protein, which acts as a substrate adaptor for the Cullin-3 (CUL3) ubiquitin ligase, which plays a role in proteasomal degradation of target proteins.^17,18^ CUL3 serves as a scaffold where components of the Cullin Ring Ligase 3 (CRL3) complex can assemble. We previously determined that phosphodiesterase 5 (PDE5) is an important target of RhoBTB1 in vascular smooth muscle cells.^15^ PDE5 plays a crucial role in the regulation of cyclic 3′,5′-monophosphate (cGMP), and consequently, the activity of nitric oxide in vascular muscle is tightly regulated by RhoBTB1-CUL3-PDE5 signaling.

RhoBTB1 is a multi-domain protein comprised of an N-terminal GTPase domain, a “sandwiched” proline-rich region, two BTB domains, and a C-terminal (CT) domain.^18^ The BTB domains are known to function as mediators of protein–protein interactions and serve as the distinctive feature of adaptor proteins that direct protein substrates towards ubiquitination via the CRL3 complex.^19^ RhoBTB1 interacts with CUL3 through its first BTB domain, and presumably to substrates through its CT domain.^20^ However, the mechanisms causing recognition of specific substrates, and the range of substrates for this and many other BTB-domain containing proteins are poorly understood. Here, we generated truncations of RhoBTB1 and assessed the domain requirements to bind to PDE5. We also identified residues required for the interaction between RhoBTB1 and CUL3 and showed they are required for CUL3-mediated degradation of PDE5, but not the binding of RhoBTB1 to PDE5. Finally, we tested the hypothesis that the same RhoBTB1 domains required to bind PDE5 could be used as bait to capture other RhoBTB1-binding proteins, some of which might be CUL3 targets. We validated several RhoBTB1 targets and showed that SET domain-containing 2 (SETD2) is regulated by proteasomal degradation via a CUL3 and RhoBTB1-dependent mechanism.

## Material and Methods

### Cell Culture

HEK293 cells were cultured in Dulbecco’s modified Eagle medium (11885084, Gibco, Thermo Fisher Scientific, USA) supplemented with 5% fetal bovine serum (10082147, Gibco, Thermo Fisher Scientific, USA) and 1% penicillin–streptomycin (15140122, Gibco, Thermo Fisher Scientific, USA) and maintained at 37°C with 5% CO_2_ as described previously.^21^ Dulbecco’s PBS (14190144, Gibco, Thermo Fisher Scientific, USA) was used to wash cells prior to harvesting with 0.05% trypsin-EDTA (25300054, Gibco, Thermo Fisher Scientific, USA).

### Transfection, Site-Directed Mutagenesis, and RNA Interference

Deletion mutants encoding Myc-tagged RhoBTB1 domains and His-tagged PDE5 were cloned in pcDNA3.1 mammalian expression vector (V79020, Invitrogen, Thermo Fisher Scientific, USA) using BamH1 and EcoRV restriction sites. CUL3 was cloned in the pcDNA3.1 mammalian expression vector using Kpn1 and EcoRV restriction sites. For proximity labeling, APEX2-NES was excised from the pcDNA3-APEX2-NES vector (49386, Addgene, USA) using Not1 and Xho1 restriction sites and re-cloned downstream of B1B2C construct using the glycine-serine linker GGSSGGSS (encoded by GGAGGCTCCTCAGGAGGTTCGTCT) in the pcDNA3.1 vector. Site-directed mutagenesis techniques were used to generate all point mutants in the B1B2C domain using the QuickChange II Site-Directed Mutagenesis Kit (200523, Agilent, USA) according to manufacturer’s instructions. Sequences of the primers used in site-directed mutagenesis, real time RT-PCR, and siRNA for RhoBTB1 are listed in Supplementary Tables S1–S3. All plasmid sequences were verified using DNA sequencing (Retrogen Inc., USA). For expression of Myc-B1B2C and His-PDE5, HEK293 cells were transfected using Lipofectamine LTX-PLUS reagent (A12621, Invitrogen, Thermo Fisher Scientific, USA) according to the manufacturer’s protocol. All siRNA duplexes were transfected using Lipofectamine RNAiMax transfection reagent (13778075, Invitrogen, Thermo Fisher Scientific, USA) according to the manufacturer’s protocol.

### Antibodies

The following antibodies were used: rabbit anti-Myc (2278S, Cell Signaling Technology, USA), rabbit anti-his (2365S, Cell Signaling Technology, USA), mouse anti-His (MA1-21315, Invitrogen, Thermo Fisher Scientific, USA), rabbit anti-CUL3 (A301-109A, Bethyl Laboratories, USA), mouse anti-GAPDH (sc-32233, Santa Cruz Biotechnology, USA), rabbit anti-SETD2 (55377-1-AP, Proteintech, USA), rabbit anti-ubiquitin (43124S, Cell Signaling Technology, USA), rabbit anti-CAMSAP1 (A302-260A, Bethyl Laboratories, USA), rabbit anti-TRAPPC9 (16014-1-AP, Proteintech, USA), rabbit anti-HMGB-1 (66525-1-Ig, Proteintech, USA), anti-rabbit HRP conjugated antibody (7074S, Cell Signaling Technology, USA), anti-mouse HRP conjugated antibody (7076S, Cell Signaling Technology, USA), His-tag mouse mAb Sepharose bead conjugate (4079S, Cell Signaling Technology, USA), Myc-tag mouse mAb Sepharose bead conjugate (3400S, Cell Signaling Technology, USA), Alexa Fluor goat anti-mouse 488 (38731, Life Technologies, USA), and Alexa Fluor 568 goat anti-rabbit (35646, Life Technologies, USA), streptavidin HRP conjugate (S911, Invitrogen, Thermo Fisher Scientific, USA).

### Immunoblot and Co-immunoprecipitation

For co-immunoprecipitation, HEK293 cells were treated with 1 μm MLN4924 (951950-33-7, Calbiochem, USA) after 16 h and 10 μm MG132 (M7449, Sigma–Aldrich, USA) after 20 h of transfection with appropriate constructs. Cell lysates were washed with ice-cold Dulbecco’s PBS, and mixed with IP lysis buffer [20 m m Tris–HCl (pH 7.5), 150 m m NaCl, 1 m m Na_2_EDTA, 1 m m EGTA, 1% Triton X-100, 2.5 m m sodium pyrophosphate, 1 mM beta-glycerophosphate, 1 m m Na_3_VO_4_, 1 µg/mL leupeptin, and 1 m m phenylmethylsulfonyl fluoride (PMSF)]. The cells were gently rotated in 1 mL centrifuge tubes for 10 min at 4°C. The supernatants were collected by centrifugation at 13 000 *g* for 10 min at 4°C. Next, 1000 μg of cell lysates were incubated with 20 μL of Myc-tag or His-tag mouse mAb Sepharose bead conjugate overnight at 4°C with gentle rotation. The beads were centrifuged at 0.8 *g* and washed 3 times (5 min each wash) with IP lysis buffer at 4°C with gentle rotation, and the immunocomplexes were dissociated by boiling in 2× sample buffer. The immunoprecipitates were then resolved by SDS-polyacrylamide gel electrophoresis (PAGE) and transferred to a nitrocellulose membrane (88018, Thermo Fisher Scientific, USA). Membranes were blocked in 3% bovine serum albumin (BSA) in 0.1% Tween-20 (P1379, Sigma–Aldrich, USA) for 1 h at room temperature. The membranes were then incubated with the appropriate primary antibody at 4°C overnight. Membranes were washed in 0.1% TBST 3 times (10 min each wash) and incubated with appropriate HRP conjugated secondary antibody for 1 h at room temperature. The membranes were again washed 3 times with 0.1% TBST, and the protein bands were visualized using enhanced chemiluminescence reagents (1705060, Bio-Rad Laboratories, USA), according to the manufacturer’s instructions.

### Ubiquitination and Cycloheximide Pulse-Chase Assay

For B1B2C mediated in vivo ubiquitination of PDE5, transfected cells were treated with MLN4924 or MG132 immediately after transfection with appropriate constructs, and the cell lysates were prepared in RIPA lysis buffer to sustain denaturing environment. The pull-down was performed, immunoprecipitates were resolved, transferred to nitrocellulose membranes, and the blots were developed using the methodology mentioned above. For the cycloheximide pulse-chase assay, the cells were co-transfected with His-PDE5 and increasing concentrations of Myc-B1B2C for 16 h and then cells were treated with 100 μm cycloheximide (C7698, Sigma–Aldrich, USA) for another 8 h. Cell lysates were prepared in RIPA buffer supplemented with protease inhibitors and 1 m m PMSF (36978, Thermo Fisher Scientific, USA). Proteins were resolved by SDS-polyacrylamide gel electrophoresis and transferred to a nitrocellulose membrane as described above. Protein levels were normalized with glyceraldehyde 3-phosphate dehydrogenase (GAPDH), and western blot images were analyzed using ImageJ and densitometric data were plotted using Prism 9.4.1 software (GraphPad, USA).

### Real-time RT-PCR

HEK293 cells were transfected with appropriate constructs for 16 h. Cells were then treated with 5 µm of Actinomycin D (SBR00013, Sigma–Aldrich, USA) for another 8 h and then suspended in Trizol reagent (15596018, Invitrogen, Thermo Fisher Scientific, USA). Total RNA was isolated, and 1 mg of RNA was reversed transcribed using 1 U of Superscript III (18080044, Invitrogen, Thermo Fisher Scientific, USA) in 20 μL reactions. Reactions were incubated at 50°C for 5 min, 4°C for 3 min, 55°C for 45 min, and finally at 70°C for 15 min for inactivation. The cDNA was diluted 1:40, and gene expression was measured using Fast SYBR Green Gene Master Mix (4385612, Applied Biosystems, Thermo Fisher Scientific, USA). The relative levels of mRNA expression were normalized to GAPDH. Data were analyzed using the 2^−ΔΔCT^ method to calculate fold-changes relative to the cells transfected with empty vector. The sequence of primers used are listed in Supplementary Table S2.

### Immunofluorescence

HEK293 cells grown in 12-well chamber slides (81201, Ibidi, Germany) were either transfected with Myc-B1B2C and His-PDE5 or co-transfected with both Myc-B1B2C and His-PDE5. At 24 h post transfection, cells were fixed with 4% paraformaldehyde (PFA; J61899.AK, Thermo Fisher Scientific, USA) and permeabilized with 0.1% Triton X-100 in blocking solution (0.5% BSA in PBS) followed by blocking for 1 h with 0.5% BSA in PBS, and then incubated overnight at 4°C with mouse monoclonal anti-His (1:100) and rabbit monoclonal anti-Myc (1:100) primary antibodies followed by 3 washes with PBS. Cells were then incubated with Alexa Fluor goat anti-mouse 488 and Alexa Fluor 568 goat anti-rabbit secondary antibodies for 1 h at room temperature followed by 3 washes with PBS. Glass slides were mounted using ProLong Gold Antifade Reagent (P10144, Invitrogen, Thermo Fisher Scientific, USA) containing DAPI. Images were acquired with a 40X objective using a confocal laser scanning microscope (LSM510; Zeiss, Oberkochen, Germany) and analyzed using the Aim 4.2 software LSM510.

### Proximity Ligation Assay (PLA)

HEK293 cells were seeded at a seeding density of 12 500 cells/well in 12-well chamber slides and grown for 2 days. After 2 days, cells were transfected with Myc-B1B2C and His-PDE5 or co-transfected with both Myc-B1B2C and His-PDE5 clones for 24 h following a published PLA protocol.^22^ The cells were washed 3 times with PBS, fixed in 4% PFA in PBS for 10 min, permeabilized by ice-cold methanol for 20 min, washed 3 times with PBS, blocked with 3% BSA in PBS, washed again with PBS, and incubated with appropriate primary antibodies for 16 h at 4°C. Cells were washed with 0.1% PBST and incubated with the appropriate amount of anti-mouse MINUS and anti-rabbit PLUS probes supplied with Duolink in situ Red Starter Kit Mouse/Rabbit (DUO92101, Sigma–Aldrich, USA), according to the manufacturer’s instructions. Finally, coverslips were mounted onto slides using ProLong Gold Antifade Reagent containing DAPI and observation fields were randomly selected using confocal laser scanning microscope (LSM510; Zeiss, Oberkochen, Germany) equipped with 40x objective and analyzed using the Aim 4.2 software LSM510. The specificity of the signal was controlled by counting the dots produced when one of the primary antibodies was omitted. For counting the fluorescent dot signals, ImageJ cell image analysis software was used. Relative number of dots/cell were plotted using Prism 9.4.1 software (GraphPad, USA).

### Protein–protein Macromolecular Docking

To investigate the possible interaction interface between RhoBTB1 and CUL3, the predicted structures of RhoBTB1 (UniProt ID: Q9DAK3) and CUL3 (UniProt ID: Q13618) were retrieved from AlphaFold (https://alphafold.ebi.ac.uk/). B1B2C domain was extracted from full-length RhoBTB1 by removing the sequence specific to GTPase domain using PyMOL software (Schrӧdinger, LLC). The models were docked using the ClusPro 2.0 webserver (https://cluspro.org/).^23^ This protein docking algorithm uses the fast Fourier transform correlation approach combined with an automatic clustering method to propose interactive surfaces with favorable free energies and outputs 10 protein–protein docking states.^24^ Protein–protein interface areas and molecular interactions were analyzed by PDBsum.^25,26^ The interaction distances between amino acid pairs and images of interacting amino acids were generated after structural analyses of the protein files using UCSF Chimera software (https://www.rbvi.ucsf.edu/chimera).^27^

### Proximity Labeling By APEX2

We followed a typical APEX2 protocol as previously described.^28^ Unless otherwise indicated, cells were treated with 1 μm MG132 for 16 h prior to harvesting to inhibit proteasomal degradation. Immediately prior to harvesting, H_2_O_2_ (H1009, Sigma–Aldrich, USA) was added to a final concentration of 1 m m, the plates were gently agitated, and incubated for exactly 1 min at room temperature. The reaction was quenched by washing 3 times with ice-cold Dulbecco’s PBS containing 5 m m Trolox (238813, Sigma–Aldrich, USA), 10 m m sodium ascorbate (PHR1279, Sigma–Aldrich, USA), and 10 m m sodium azide (190385000, Thermo Fisher Scientific, USA). Cells were then lysed in RIPA lysis buffer supplemented with 1 m m PMSF, protease inhibitor cocktail (1861281, Thermo Fisher Scientific, USA), 5 m m Trolox, 10 m m sodium ascorbate, and 10 m m sodium azide, and then centrifuged at 15 000 × *g* for 10 min at 4°C. Biotinylated protein was purified by incubation with streptavidin beads (88817, Thermo Fisher Scientific, USA) overnight with gentle rotation at 4°C. The following buffers were used to thoroughly wash the beads to remove nonspecific binders: twice with RIPA lysis buffer, once with 1.0 M KCl (P41025, RPI Research Products Int., USA), once with 0.1 M Na_2_CO_3_ (L13098.36 Thermo Fisher Scientific, USA), once with 2.0 M urea (15505035, Thermo Fisher Scientific, USA) in 10 m m Tris (pH 8.0; T5941, Sigma–Aldrich, USA), and twice with RIPA lysis buffer. To examine the activity of the APEX2 tagged fusion construct using western blot, the biotinylated proteins were eluted by boiling the beads in 3× protein loading buffer containing 20 m m DTT (D9779, Sigma–Aldrich, USA) and 2m m biotin (B4501, Sigma–Aldrich, USA) for 10 min, then resolved on 10% SDS-PAGE and transferred to a nitrocellulose membrane. Membranes were blocked in 3% BSA in 0.1% Tween-20 (P1379, Sigma–Aldrich, USA) for overnight at 4°C. Immunoblotting was done using streptavidin HRP conjugate for 1 h at room temperature. The membrane was washed with TBST again, and the biotinylation was visualized using enhanced chemiluminescence reagents according to the manufacturer’s instructions. For mass spectrometry, the suspension buffer was removed from the pulldown beads using a magnetic rack. Beads were resuspended in 100 µL of 40% Invitrosol (MS1000, Invitrogen, Thermo Fisher Scientific, USA) and 100 m m ammonium bicarbonate (09830, Fluka, Switzerland). Cysteines were reduced in 5 m m TCEP (646547, Sigma–Aldrich, USA) for 30 min at 37°C and alkylated with 10 m m iodoacetamide (100351, MP Biomedicals, USA) for 30 min at 37°C. Biotinylated proteome pulldown was digested directly on streptavidin beads overnight with 5 µg of trypsin (PR-V5113, Promega) at 37°C followed by cleanup using the SP2 method. Thermo Scientific Pierce Peptide Retention Time Calibration Mixture (88321, Thermo Fisher Scientific, USA) was added at 4 n m concentration during dilution of the samples to 20 ng/µL.

### Mass Spectrometry Analysis

Each sample was analyzed on a Thermo Scientific Orbitrap Fusion Lumos MS via 3 technical replicate injections using a data-dependent acquisition (DDA) HCD MS2 instrument, using the parameters outlined in Supplementary Table S4. The technical replicates were blocked, and each block was randomized. Pooled QCs, which is a mixture of all the samples being analyzed, were analyzed at the start, end, and in between each sample. MS data were analyzed using Proteome Discoverer 2.4 (Thermo) platform as outlined in the Supplementary Table S5. Protein identifications were filtered to include only those proteins identified by two or more unique peptides identified. Mass spectrometry proteomics data have been deposited to the Proteome Xchange Consortium via the PRIDE partner repository with the dataset identifier PXD043245 and 10.6019/PXD043245.

### Statistical Analysis

Results are expressed as the mean of independent experiments ± SEM. The number of repeats (*n*) used in each experiment is indicated in the figures and text. Statistical significance was determined using one-way ANOVA using Prism 9.4.1 software (GraphPad, USA). A *P*-value less than 0.05 was considered statistically significant and indicated with an asterisk in figures.

## Results

### PDE5 Interacts With B1B2C Domain of RhoBTB1

**Figure 1.**
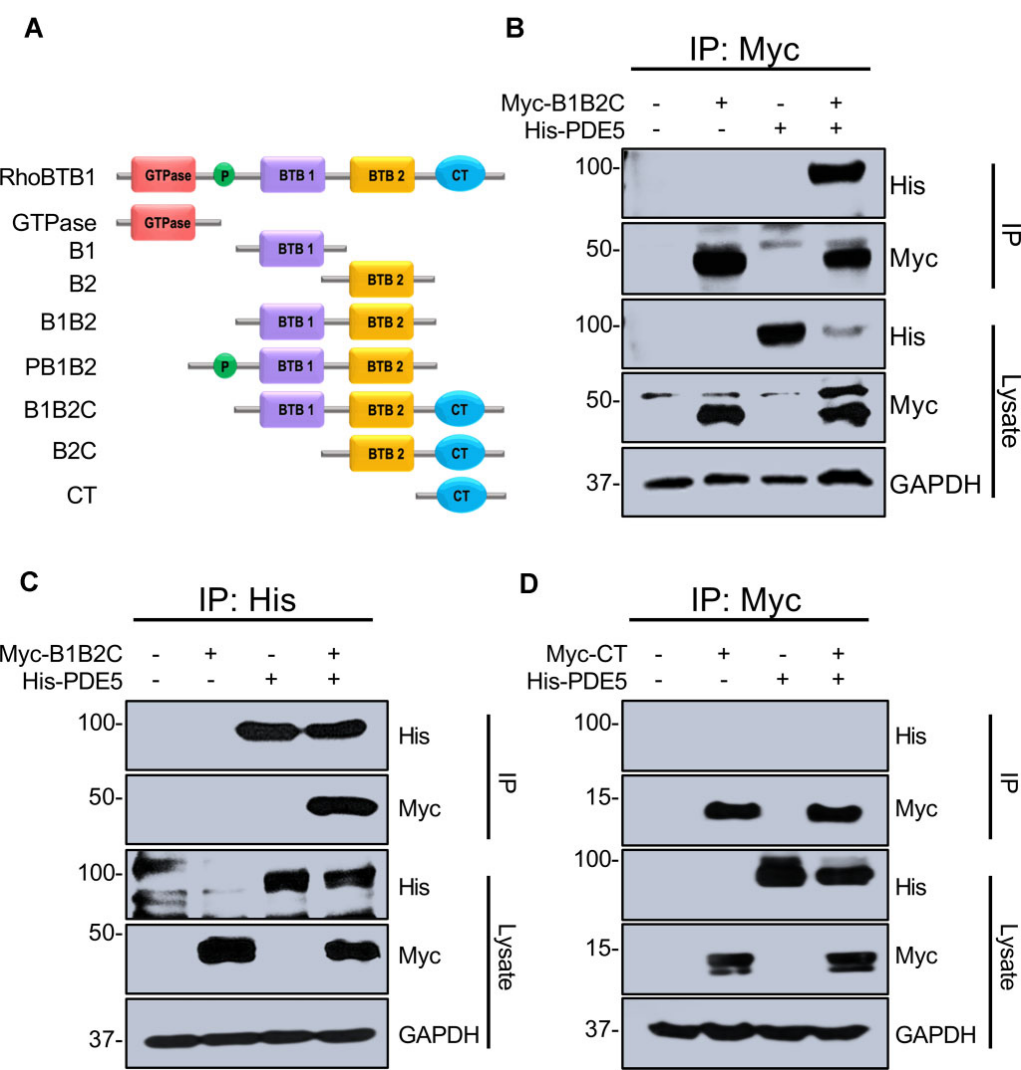
RhoBTB1 domain architecture and association with PDE5. (A) RhoBTB1 is comprised of four structurally and functionally different domains: GTPase, Proline rich (P), BTB1 (B1), BTB2 (B2), and C-terminal (CT). An N-terminal Myc-epitope tag was added to each construct. (B) HEK293 cells were transfected with Myc-B1B2C and His-PDE5 for 16 h and treated with 1 μm MLN4924 after 16 h and 10 μm MG132 after 20 h of transfection. Total transfection time was 24 h. Whole-cell extracts were prepared and immunoprecipitated with the indicated antibodies, and immunoprecipitates were resolved by SDS-PAGE and immunoblotted with the indicated antibodies. (C) Reciprocal co-immunoprecipitation of the same proteins in panel B. (D) HEK293 cells were transfected with Myc-CT (C-terminal domain without the B1 and B2 domains) and His-PDE5 and treated as above. IP and lysates are indicated. Molecular weight markers were transferred from the original blots. These data are representative of two independent experiments.

RhoBTB1 consists of five distinct domains: (a) an atypical GTPase domain, which may not exhibit GTPase activity, (b) a proline rich domain, (c) two BTB1 domains characteristic of those needed to associate with CUL3, and (d) a C-terminal domain (Figure 1A). RhoBTB1 is an adaptor protein designed to deliver specific substrates to the CUL3 ring ubiquitin ligase complex. PDE5 is the only known and validated substrate for RhoBTB1. To identify the minimal domain(s) on RhoBTB1 required to interact with PDE5, we generated a series of truncation mutants of RhoBTB1 carrying a single or multiple domains (Figure 1A). We performed a series of co-immunoprecipitation experiments in HEK293 cells transfected with Myc-tagged domains of RhoBTB1 and full length His-tagged PDE5. Truncated domains of RhoBTB1 were immunoprecipitated and analyzed by western blotting for the presence of PDE5 in the immune complexes.

**Figure 2.**
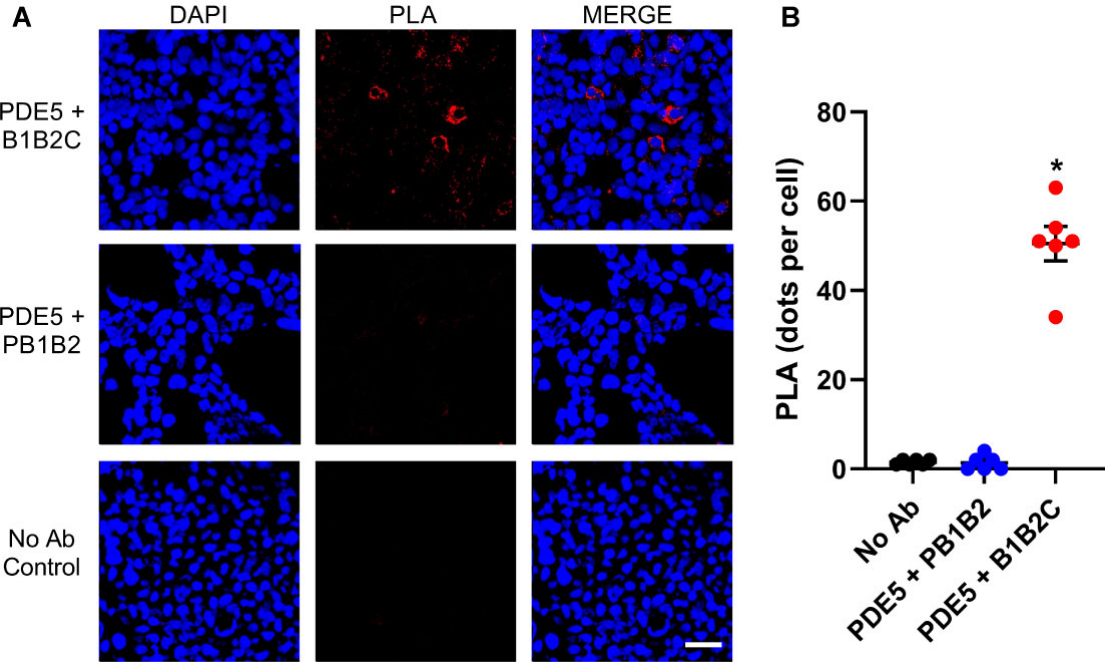
PLA for B1B2C and PDE5. (A) HEK293 cells were transfected with His-PDE5 and Myc-B1B2C or His-PDE5 and Myc-PB1B2 for 16 h and transfected cells were then treated with 1 μm MLN4924 after 16 h and 10 μm MG132 after 20 h of transfection. Anti-His and anti-Myc antibodies are specifically recognized by complementary oligonucleotide probes rabbit PLUS and mouse MINUS. When two interacting proteins are close enough (<40 n m), hybridization of the probes occurs, which are then amplified resulting in the incorporation of fluorescent nucleotides giving a red positive PLA signal (puncta). Fluorescent puncta generated upon the proximity of PDE5 and B1B2C confirms the interaction. No PLA signal was reported in negative no-antisera control establishing the specificity of interaction. The scale bar is 50 μm. (B) Graph shows the quantification of PLA signal. *P < 0.05 by one-way ANOVA.

Single or combination domains, including GTPase, B1B2, PB1B2, B1, and B2 lacking the CT domain failed to interact with PDE5 despite evidence for their expression in lysates (Supplementary Fig. S1, data for B1 and B2 domains alone are not shown). However, the addition of the CT domain to the two BTB domains (B1B2C) resulted in co-IP with PDE5 (Figure 1B). Reciprocal co-immunoprecipitation analysis also confirmed the interaction between PDE5 and B1B2C (Figure 1C). Notably, the CT domain could not interact with PDE5 on its own (Figure 1D). Collectively, these findings suggested the CT domain is necessary but not sufficient to mediate binding to PDE5. To validate the specificity of the B1B2C:PDE5 interaction quantitatively, in situ PLA was performed. Consistent with the results obtained by co-immunoprecipitation, we obtained a strong PLA signal in the groups co-transfected with Myc-B1B2C and His-PDE5 (Figure 2). No significant PLA signal was detected in the negative control groups either stained without primary antibody or transfected with RhoBTB1 without the CT domain (PB1B2).

### Ectopically Expressed B1B2C Negatively Regulates Intracellular PDE5

**Figure 3.**
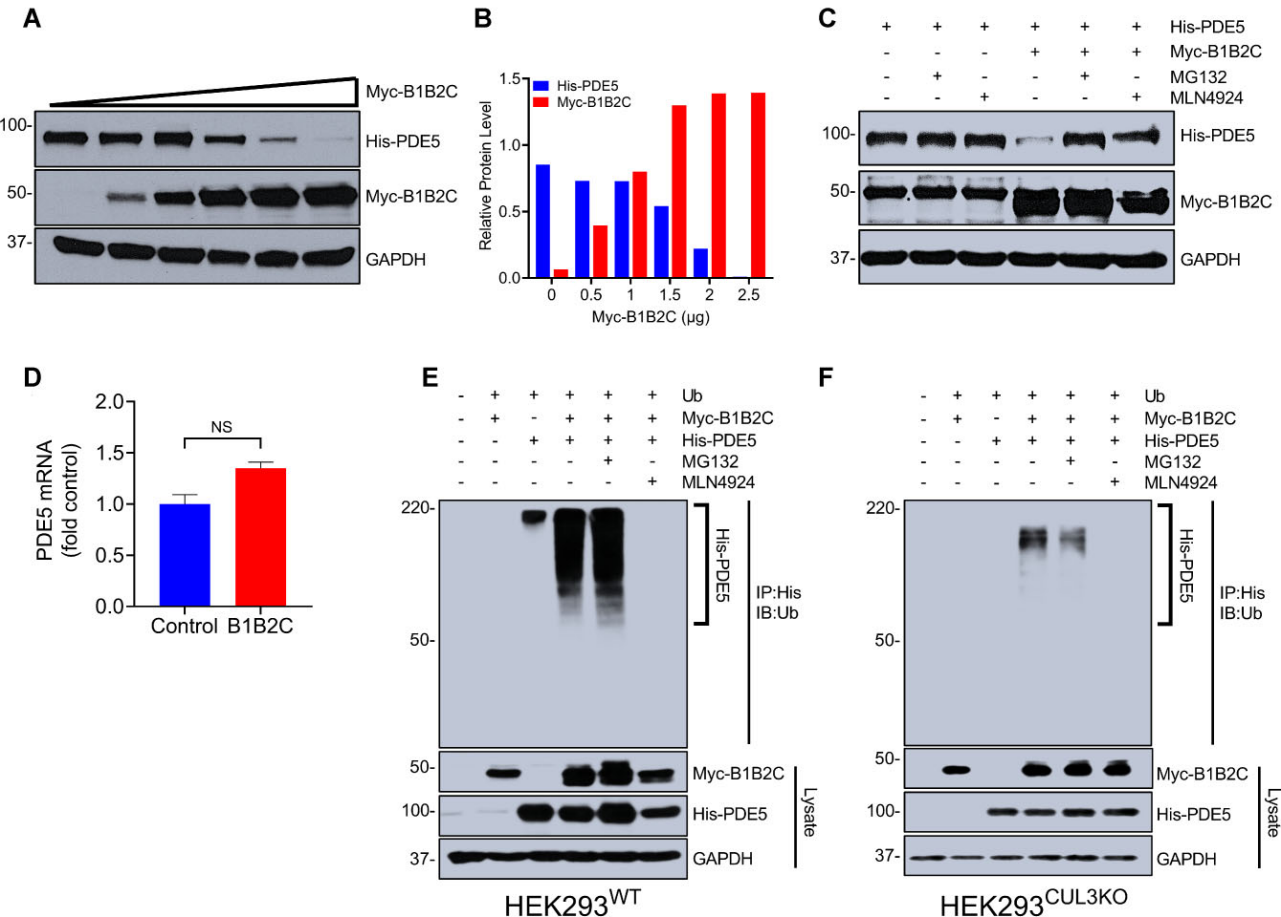
B1B2C is sufficient to regulate PDE5 through ubiquitination. (A) HEK293 cells were transfected with the empty vector or ramped increases in Myc-B1B2C and His-PDE5 for 16 h, after which cells were treated with 100 μm cycloheximide for 8 h. Cells were harvested, the whole-cell lysates were collected, and extracts were immunoblotted with the indicated antibodies. (B) Relative levels of Myc-B1B2C and PDE5 were quantified using ImageJ software. Data are average of duplicate experiments. (C) HEK293 cells were transfected with the empty vector or Myc-B1B2C and His-PDE5 for 16 h. Cells were treated with 100 μm cycloheximide and some cells were treated with 1 μm MLN4924 or 10 μm MG132. Whole-cell extracts were resolved by SDS-PAGE and immunoblotted with the indicated antibodies. Data are representative of two independent experiments. (D) Real-time PCR was performed to quantify PDE5 mRNA in cells also transfected with either empty vector of Myc-B1B2C. Data are presented as mean ± SEM (*N* = 3). There was no significance by *t*-test. (E) and (F) Wild-type HEK293 cells (E) and CRISPR/Cas-9 edited CUL3 knockout HEK293 cells (F) were transfected with the empty vector or Myc-B1B2C and His-PDE5 with expression vector encoding ubiquitin for 24 h. Immediately after the transfection, cells were treated with 1 μm MLN4924 or 10 μm MG132. Whole-cell lysates were collected, and immunoprecipitation was performed under denaturing conditions. Under these conditions, B1B2C is not pulled down by PDE5. Immune complexes were probed with the indicated antisera. IP and lysates are labeled. Molecular weight markers are translated from original blots. These data are representative of two independent experiments.

A previous study from our group showed that RhoBTB1 acts as a substrate adaptor that delivers PDE5 to the CRL3 complex for its degradation, and thus, RhoBTB1 controls vascular tone by regulating nitric oxide/cGMP signaling.^15^ To determine whether B1B2C, the minimal region required to bind to PDE5, is also sufficient to regulate intracellular PDE5 levels, we transiently expressed increasing concentrations of B1B2C in HEK293 cells. The relative level of His-tagged PDE5 protein expression notably decreased as the amount of B1B2C increased in the presence of cycloheximide, an inhibitor of protein synthesis (Figure 3A and B). Also, in the presence of cycloheximide, the decrease in PDE5 caused by B1B2C overexpression was blocked by the proteasomal inhibitor MG132 suggesting a role for the proteasome (Figure 3C). Similarly, the B1B2C-mediated decrease in PDE5 was blocked by MLN4924, a neddylation inhibitor that acts as a pan-Cullin inhibitor. There was no decrease in PDE5 protein in presence of PB1B2 domain lacking the CT domain (Supplementary Figure S2). Importantly, there was no change in PDE5 mRNA in the presence or absence of the B1B2C domain, consistent with a post-translation mechanism (Figure 3D). Thus, we concluded that the B1B2C domains are sufficient on their own to regulate the intracellular levels of PDE5, and that the GTPase and Proline-rich domains are dispensable.

### B1B2C Promotes Polyubiquitination of PDE5

BTB-domain containing proteins can interact and promote the polyubiquitination of their substrates through the CRL3 complex.^29^ Therefore, we asked if the decrease in PDE5 mediated by B1B2C occurs through ubiquitination of PDE5. His-tagged PDE5 was co-transfected with ubiquitin with either empty vector or Myc-tagged B1B2C in HEK293 cells. Immunoblot analysis revealed the extensive ubiquitination of PDE5 only in the presence of B1B2C (Figure 3E). Consistent with a role for CUL3, ubiquitination of PDE5 was blocked by MLN4914. To further delineate the role of CUL3 in B1B2C aided ubiquitination of PDE5, we expressed PDE5 and ubiquitin with either empty vector or B1B2C in a previously established CUL3-deficient HEK293 cell line made by CRISPR/Cas-9. In the absence of CUL3, PDE5 ubiquitination was drastically decreased in cells transfected with B1B2C. (Figure 3F). Interestingly, the ubiquitination of PDE5 was not completely abolished in CUL3 knockout HEK293 cells suggesting the possible involvement of alternative ubiquitination enzymes. Taken together, we concluded that B1B2C is responsible for ubiquitination of PDE5 followed by its degradation via CUL3-dependent ubiquitin-proteasomal pathway, and the N-terminal GTPase and Proline-rich domains are dispensable.

### Protein–protein Interaction Revealed CUL3 Binding Hotspots on B1B2C

Since the results above showed that both CUL3 and the B1B2C domain of RhoBTB1 were critical for PDE5 ubiquitination, we studied the specific amino acids in B1B2C required for its binding to CUL3. We performed macromolecular protein–protein docking between CUL3 and B1B2C. Following protein–protein docking, the docking poses were filtered based on cluster with maximum numbers to visualize the interface between CUL3 and B1B2C. Because PDBsum identifies protein–protein interactions as chain–chain interactions, we annotated the CUL3 and B1B2C domains as chains A and B, respectively.^30^ A PDBsum analysis of CUL3 and B1B2C reported an interacting interface comprising of 24 amino acid residues and the potential distance between them (Supplementary Figure S3). B1B2C–CUL3 complex formation is mediated by hydrogen bonds, electrostatic, and non-bonded interactions (Supplementary Table S6). Further structural investigation revealed that Pro^353^ (numbering based on native RhoBTB1) on B1B2C interacts with Arg^546^ on CUL3 with the help of hydrogen bond and non-bonded interactions. Similarly, the interaction between Ser^363^ on B1B2C and Arg^529^ on CUL3 occurs by hydrogen bond and non-bonded interactions. In addition, interactions were also noticed for Ser^363^ on B1B2C and Cys^522^, Asn^523^, and Ile^524^ on CUL3.

**Figure 4.**
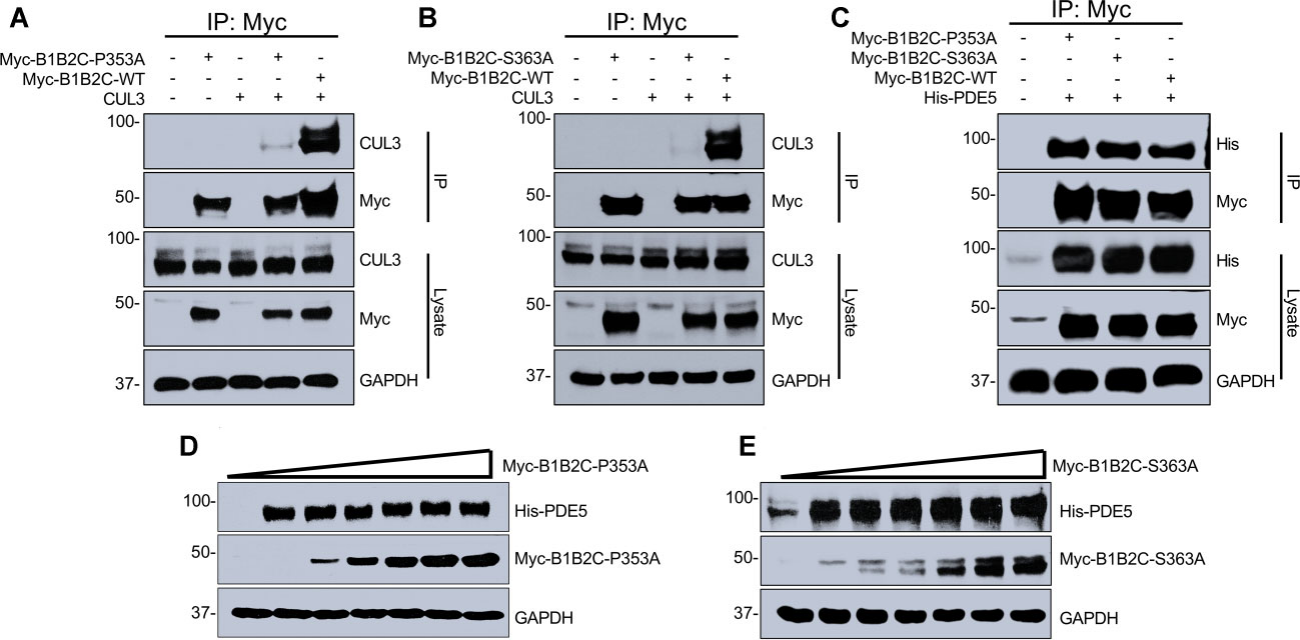
RhoBTB1 mutants fail to associate with CUL3 and to degrade PDE5. (A) and (B) HEK293 cells were transfected either with (A) Myc-B1B2C-P353A or (B) Myc-B1B2C-S363A and CUL3. Cells were treated with 1 μm MLN4924 16 h after transfection, and 10 μm MG132 20 h after transfection. Whole-cell extracts were prepared and immunoprecipitated with the indicated antibodies. Immunoprecipitates were resolved by SDS-PAGE and immune complexes were probed with the indicated antisera. Co-immunoprecipitation of CUL3 with wildtype B1B2C (Myc-B1B2C-WT) is shown as a control. IP and lysates are labeled. (C) HEK293 cells were transfected with Myc-B1B2C-P353A or Myc-B1B2C-S363A and His-PDE5. Cells were then treated with 1 μm MLN4924 16 h after transfection and 10 μm MG132 20 h after transfection. Whole-cell extracts were prepared and immunoprecipitated with the indicated antibodies. (D) and (E) HEK293 cells were transfected with the empty vector or increasing amounts of Myc-B1B2C-P353A (D) or Myc-B1B2C-S363A (E) and with His-PDE5. Cells were then treated with 100 μm cycloheximide for 8 h. Cells were harvested, the whole-cell lysates were collected, and extracts were immunoblotted with the indicated antibodies. Molecular weight markers were transferred from the original blots. Data are representative of two independent experiments.

We next tested whether the specific interacting residues identified in the CUL3–B1B2C complex are the major determinants of binding specificity using site directed mutagenesis and co-immunoprecipitation assays. We hypothesized that substituting the CUL3 binding residues on B1B2C would hamper its binding with CUL3, and therefore CUL3-dependent degradation of PDE5. Results of co-immunoprecipitation revealed that the interaction of B1B2C mutants Pro353Ala and Ser363Ala with CUL3 was markedly decreased compared to wild-type B1B2C and CUL3 despite exhibiting similar levels of expression in the lysates (Figure 4A and B). Interestingly, despite the decreased binding of the mutants to CUL3, their binding to PDE5 was preserved (Figure 4C). Consistent with decreased binding to CUL3, both mutants were unable to mediate proteasomal degradation of PDE5 (Figure 4D and E, Supplementary Figure S4). Therefore, multiple sites along the interface are required for stabilization of the B1B2C and CUL3 complex.

### Ascorbate Peroxidase 2 (APEX2) Labeling Resolves B1B2C Interacting Proteome

Next, we identified other interacting partners in the RhoBTB1-CUL3 pathway. We employed the APEX2 labeling system utilizing an APEX2-tagged B1B2C (B1B2C–APEX2) fusion construct. The outline of the experiment is depicted in Supplementary Figure S5A. First, we showed that B1B2C–APEX2 was expressed in HEK293 cells as immunoblotting confirmed the expression of the fusion construct at the expected molecular weight (Supplementary Figure S5B). Second, HEK293 cells transfected with B1B2C–APEX2 were subjected to biotinylation involving treatment of cells with biotin-phenol and a fractional pulse of H_2_O_2_. Immunoblotting with streptavidin-HRP conjugate indicated robust biotinylation of numerous proteins in the presence of the B1B2C–APEX2 construct, biotin-phenol, and H_2_O_2_ demonstrating that the construct is functional (Supplementary Figure S5C). Consistent with this, no biotinylation was detected in non-transfected cells treated with either biotin-phenol or H_2_O_2_ or both, or in transfected cells treated individually with of biotin-phenol or H_2_O_2_.^31^ To validate the interaction capability of B1B2C–APEX2, we co-expressed B1B2C–APEX2 (as a Myc-tagged construct) and PDE5 in HEK293 cells. PDE5 was immunoprecipitated and analyzed by western blotting for the presence of B1B2C–APEX2 in the immunoprecipitate. Results of co-immunoprecipitation revealed that the interaction of B1B2C–APEX2 with PDE5 was preserved, validating that the APEX2 tag does not interfere with the interaction with PDE5 (Supplementary Figure S5D).

**Figure 5.**
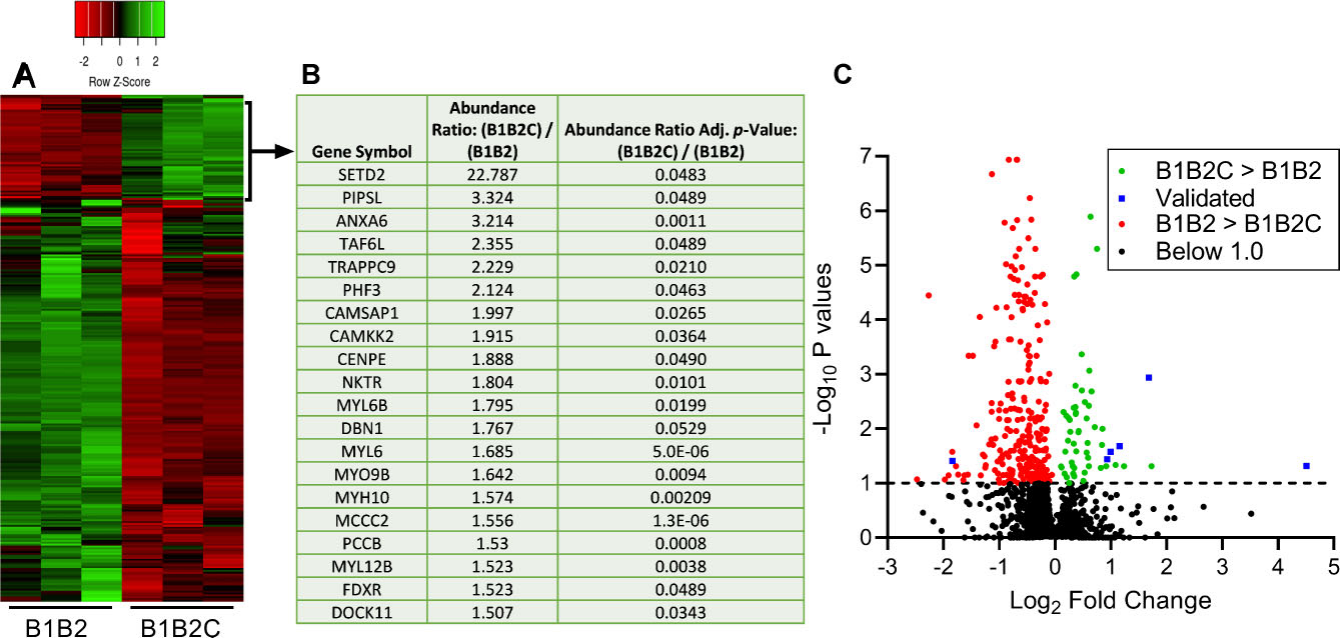
Profiling of the B1B2C interacting proteome in HEK293 cells. (A) Heatmap with hierarchical clustering demonstrating the differential interaction of proteome with B1B2C and B1B2 domains of RhoBTB1. HEK293 cells expressing B1B2–APEX2 were used to detect background biotinylation. (B) Twenty proteins were selected by applying a cut-off interaction B1B2C/B1B2 > 1.5-fold. (C) Volcano plot showing proteins, which interacted better with B1B2C than B1B2 (green/blue) and proteins, which interacted better with B1B2 than B1B2C (red). Proteins in black were non-significantly different in binding to B1B2C or B1B2. The proteins labels in blue represent those which were validated by co-IP in  Figure 6.

To rule out the surplus labeled proteome, we performed a contemporaneous screen using APEX2-tagged B1B2 (lacking the C terminal region) in HEK293 cells. Thus, comparing proteins identified by B1B2C–APEX2 versus B1B2–APEX2 allowed us to specifically capture the proteome interacting with C-terminal region of RhoBTB1. The biotinylated proteome was enriched using streptavidin beads and identified by mass spectrometry. Overall, out of a total of 2448 enriched proteins across 6 samples (3 technical replicates across two conditions: B1B2 and B1B2C), 268 proteins were selected based on statistical significance (Benjamini–Hochberg *P*-value < 0.05, Figure 5A and Supplementary Table S7). Then, we selected 20 candidate proteins based on fold enrichment (Figure 5B). These proteins were found to be enriched more with B1B2C than B1B2 (abundance ratio B1B2C/B1B2: > 1.5-fold). A volcano plot graphically represents those proteins found to interact better with B1B2C (green), B1B2 (red), or below the level of statistical significance (black, Figure 5C). Proteins chosen for further analysis are shown in blue.

These differentially interacting proteins were further analyzed for their molecular processes and enriched pathways using the ShinyGO tool (version 0.76.3). In ShinyGO analysis, two pathways (nodes) are connected if they share 20% (default) or more genes. Darker nodes are more significantly enriched gene sets. Bigger nodes represent larger gene sets while thicker edges represent more overlapped genes. Most of the regulated pathways in our data set were related to calcium signaling, muscle contraction, nucleotide binding, and cytoskeletal remodeling (Supplementary Figure S6). This is consistent with our previous report that RhoBTB1 regulated arterial stiffness through the cytoskeleton organization.^16^

## Physical Interactions of B1B2C Interacting Proteome Were Confirmed By Co-IP

**Figure 6.**
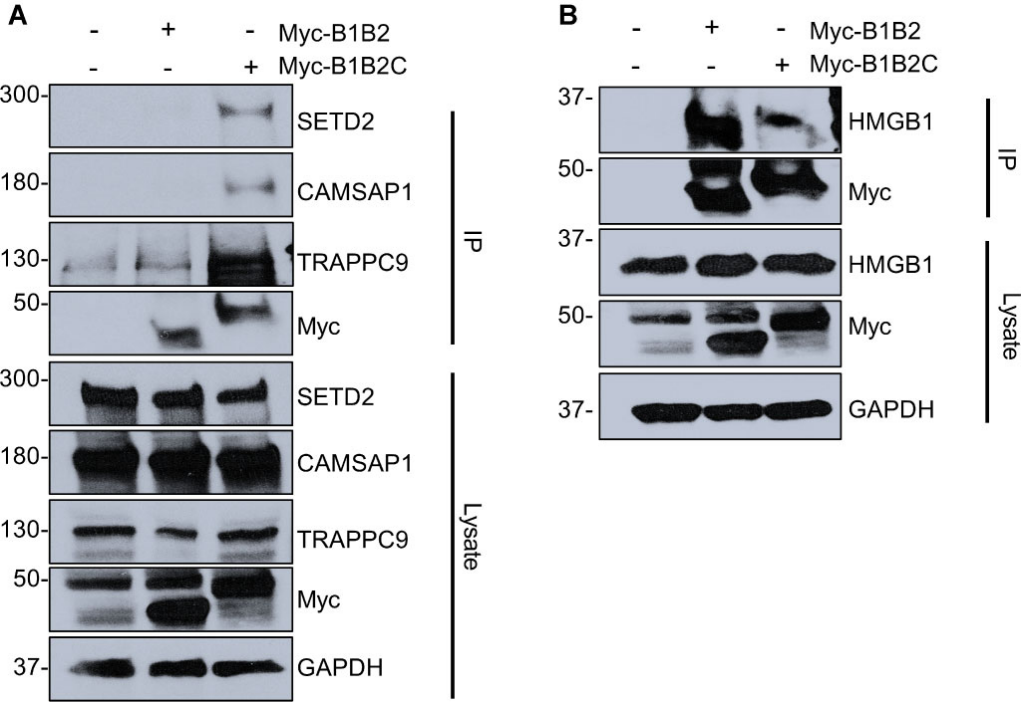
Validation of RhoBTB1 binding proteins by co-immunoprecipitation. (A) HEK293 cells were transfected with Myc-B1B2C and Myc-B1B2 individually for 16 h. Immunoprecipitates were resolved by SDS-PAGE and were immunoblotted for endogenous proteins that were identified to bind B1B2C greater than B1B2 (SETD2, CAMSAP1, TRAPPC9). (B) HEK293 cells were transfected with Myc-B1B2C and Myc-B1B2 individually for 16 h. Immunoprecipitates were resolved by SDS-PAGE and were immunoblotted for endogenous HMGB1, which was identified to bind B1B2 greater than B1B2C. All transfected cells were treated with 1 μm MLN4924 after 16 h and 10 μm MG132 after 20 h of transfection. Whole-cell extracts were prepared and immunoprecipitated with the indicated antibodies, and IP and lysates are labeled. Molecular weight markers were transferred from the original blots.

We next validated the interaction of several of the top scored interactors detected by the proximity-dependent labeling approach: SET Domain Containing 2 (SETD2), Calmodulin Regulated Spectrin Associated Protein 1 (CAMSAP1), and Trafficking Protein Particle Complex Subunit 9 (TRAPPC9) for their physical interaction with B1B2C and B1B2 using Co-IP. We found each of these proteins interacted with B1B2C better than B1B2 (Figure 6A). Similarly, two other candidates (Annexin A6, ANXA6 and Calcium/calmodulin-dependent Protein Kinase Kinase 2, CAMKK2) also interacted with B1B2C (data not shown). As an internal control, we selected High Mobility Group Box 1 (HMGB1), which interacted better in the APEX2 screen with B1B2 when compared to B1B2C (B1B2/B1B2C abundance ratio: 1.57 versus 0.44 and *P*-value = 0.039). Consistent with the mass spectrometry data, HMGB1 was found to interact with B1B2 greater than B1B2C (Figure 6B). Therefore, the high throughput mass spectrometry data corroborated our co-immunoprecipitation findings. Given this, we reasoned that SETD2, ANXA6, CAMKK2, CAMSAP1, and TRAPPC9 may interact with RhoBTB1.

## Cellular Level of SETD2 Is Regulated By RhoBTB1 and CUL3

**Figure 7.**
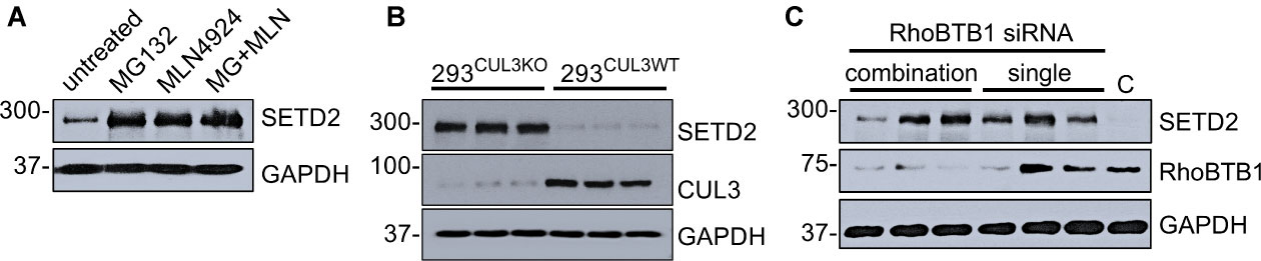
Validation of SETD2 as a target of RhoBTB1-mediated regulation. (A) The steady state levels of endogenous SETD2 were examined under pharmacological inhibition of CUL3-proteasomal pathway. HEK293 cells were treated with MG132 (5 μm) and MLN4924 (1 μm) individually or in combination for 48 h. (B) The levels of SETD2 expression were examined in wildtype and in CRISPR/Cas-9 edited CUL3 knockout HEK293 cells. (C) SETD2 expression was examined in wildtype HEK293 cells transfected with different siRNAs or combinations of siRNA targeting RhoBTB1 for 72 h. Blots were probed with indicated antisera. GAPDH was used as loading control. Data are representative of two independent experiments.

Evidence from previous studies suggested that SETD2 is degraded by the proteasomal pathway.^32,33^ Given this, we hypothesized that the cellular levels of SETD2 might be controlled by RhoBTB1 and CUL3. First, we showed that the level of SETD2 protein was markedly increased in response to proteasomal (MG132) or Cullin (MLN4914) inhibition (Figure 7A). Second, the level of SETD2 was markedly increased in HEK293^CUL3KO^ cells, suggesting its regulation is through a CUL3-dependent mechanism (Figure 7B). Third, we used a siRNA approach to knockdown the expression of RhoBTB1 in HEK293 cells. The knockdown of RhoBTB1 mRNA was variable with single siRNAs knocking down RhoBTB1 mRNA between 40%–80%, and combination or two or more siRNAs by about 70%–90% (data not shown). Whereas the effect on SETD2 mRNA was 2-fold or less (generally decreased), the level of SETD2 protein (on average) increased as RhoBTB1 decreased (Figure 7C). Taken together, our data suggested that SETD2 protein expression was regulated by RhoBTB1 and CUL3.

## Discussion

We previously reported that RhoBTB1 is a PPARγ target gene involved in regulating arterial BP, vascular function, and arterial stiffness.^12,16^ One of the mechanisms by which this occurs is that RhoBTB1 serves as a substrate adaptor for CUL3-mediated ubiquitination and proteasomal degradation of PDE5.^15^ The CUL3-RhoBTB1-PDE5 regulatory circuit is critical for the regulation of vasoconstriction and vasodilation because PDE5 controls the level of cGMP in vascular smooth muscle, the downstream mediator of endothelium-derived nitric oxide.^6,18^ We hypothesized that RhoBTB1 would exhibit a range of substrates because: (a) other BTB-domain containing proteins act as adaptors for CUL3-mediated regulation,^34^ (b) other BTB-domain containing proteins have multiple substrates,^35^ and (c) RhoBTB1 is ubiquitously expressed.^36^

We further hypothesized that the binding site for PDE5 on RhoBTB1 would be similar to the binding sites for other substrates on RhoBTB1. We therefore sought to identify the minimal RhoBTB1:PDE5 interaction domain to employ it as a bait to capture and identify other RhoBTB1-CUL3 substrates. Thus, this investigation made three fundamental discoveries regarding the biology of RhoBTB1. First, we demonstrated that the B1B2C domain of RhoBTB1 is the minimal binding region for PDE5. Second, we identified key residues required for the interaction between RhoBTB1 and CUL3, which are dispensable for the binding of RhoBTB1 to PDE5 but are required for PDE5 turnover. Third, using B1B2C as bait in APEX2-based proximity ligation, we identified interacting partners of RhoBTB1 and validated several of them by co-immunoprecipitation. We further identified that one of them, SETD2, is regulated by the RhoBTB1-CUL3 pathway.

First, we demonstrated that RhoBTB1 binds to PDE5 utilizing its B1B2C domain. Interestingly, the B1B2C domain was sufficient, in the absence of the N-terminal GTPase and proline-rich domains, to deliver PDE5 to the CRL3 complex for proteasomal degradation. This is interesting on its own as it suggests that the N-terminal portion of the protein is dispensable for its role as a substrate adaptor for PDE5 degradation, and perhaps other substrates for CUL3-mediated ubiquitination. Moreover, this suggests that RhoBTB1 may be a multifunctional protein, which requires its N-terminus for other functions. RhoBTB1 is a member of a family of atypical GTPases, but contains mutations in key residues, which may abolish or limit its GTPase activity, although this remains controversial.^37,38^ Like other proteins with a proline-rich domain, RhoBTB1 may play a role in signal transduction or regulation of the cytoskeleton.^39^ Indeed, we showed that RhoBTB1 may regulate, in a manner which may or may not require its role in the CRL3 complex, the state of actin polymerization in vascular smooth muscle cells.^16^ RhoBTB1 has also been reported to interact with Rho kinases to inhibit cellular invasion.^40^ The interaction between RhoBTB1 and Rho kinases has implications for vascular function. RhoBTB1 has also been reported to regulate the Golgi apparatus and bone resorption, among others.^18,41,42^

Truncations of RhoBTB1 lacking the CT domain were unable to bind and proteolytically degrade PDE5. Thus, one of the important conclusions from this study is that the CT domain is essential for maintaining the functionality of RhoBTB1, at least with respect to ubiquitination and degradation of PDE5, but is not sufficient to bind to PDE5 on its own. Similarly, the CT domain was required to mediate an interaction between RhoBTB1, and other RhoBTB1-binding proteins identified by APEX2-mediated proximity ligation, including SETD2, CAMSAP1, TRAPPC9, ANXA6, and CAMKK2. In the case of SETD2, inhibition of either CUL3 or RhoBTB1 caused an increase in SETD2 abundance. One of the remaining questions is whether the interactions mediated by the N-terminal of RhoBTB1 coordinate with its C-terminal role as a substrate adaptor for CUL3. Interestingly, the family of RhoBTB proteins have been reported to exhibit autoinhibition by interaction of the N-terminus with the first BTB domain (BTB1), which becomes relieved by binding to CUL3 and associated proteins.^35^

CUL3 plays a key role by acting as a scaffold in the CRL3 complex, and hence, it aids in assembling the “substrate” proteins through BTB-domain containing proteins, such as RhoBTB1. Therefore, in the second phase of our study, we focused on deciphering the underlying molecular mechanisms involved in the binding of RhoBTB1 to CUL3. Macromolecular protein–protein docking revealed that Pro^353^ and Ser^363^ amino acid residues on B1B2C are crucial for its binding to CUL3. Notably, point mutations of the CUL3 binding sites on the B1B2C domain of RhoBTB1 preserved normal binding to PDE5, but were unable to facilitate the proteasomal degradation of PDE5 despite exhibiting similar expression levels. We therefore conclude that specific domains on RhoBTB1, CUL3, and PDE5 are all required for the formation and functionality of a productive CRL3 complex regulating PDE5.

RhoBTB1 is transcriptionally regulated by PPARγ. This is interesting because mutations in PPARγ cause human HTN.^7^ RhoBTB1 has also been identified in a genome wide association study to be associated with diastolic BP, and to be associated as an interacting loci in a study of over 1 million subjects.^43,44^ Expression of RhoBTB1 is markedly decreased in mice carrying a transgene expressing dominant negative mutation in PPARγ.^12^ Expression of over 100 other PPARγ-target genes were also decreased in these mice. Remarkably, the restoration of RhoBTB1 on its own by a PPARγ-independent mechanism ameliorated adverse cardiovascular phenotypes in animals carrying a dominant-negative PPARγ mutation.^15^ RhoBTB1 is also downregulated in models of angiotensin-II mediated HTN and restoration of RhoBTB1 rapidly reverses arterial stiffness.^16^ This data clearly implicated vascular smooth muscle PPARγ-RhoBTB1-CUL3-PDE5 as an important regulator of arterial BP. However, it is notable that RhoBTB1 is ubiquitously expressed with the highest level of transcripts in skeletal muscle, kidney, placenta, and testis.^35^ Single cell sequencing reveals detectable RhoBTB1 in a diverse set of cell types, including adipocytes, macrophages, excitatory neurons, ciliated cells of the respiratory tract, endometrial stromal cells, proximal tubular cells, placental syncytiotrophoblasts, and a variety of vascular cells types. We therefore must consider that like other BTB-domain containing proteins, which act as CUL3 adaptors, RhoBTB1 may have more than one substrate, perhaps many.

To investigate this possibility, we chose to utilize a proximity labeling method that involved the use of APEX2-fusion proteins in HEK293 cells, followed by proteomic profiling with mass spectrometry. We took an approach to identify proteins, which interacted with B1B2C greater than B1B2 (lacking the CT) to avoid identifying proteins, which may bind through the BTB domains, an evolutionarily conserved module, which is involved in protein–protein interactions. These domains are found in proteins that act as substrates for CUL3 but are also found in some zinc finger transcription factors and ion channels.^34^ This was important as over 2400 interactions were identified in the screen, many of which overlapped between B1B2 and B1B2C. In retrospect, it might have been valuable to use B1B2C mutants, which prevented interaction with CUL3 but preserved interaction with PDE5. However, the CUL3-RhoBTB1 experiments were performed concurrently with the APEX2 experiments. It remains unclear if that would prevent binding to other BTB-domain containing proteins.

Given that APEX2 labels not only direct interactors but also proteins in the proximity of the bait, we were not surprised to see many potential targets. Indeed, we expected to observe many false positives. However, the criteria of focusing on proteins that were detected with B1B2C greater than B1B2 allowed us to prioritize which proteins co-immunoprecipitated with B1B2C. Our validation studies also ruled out a proteome that might be reacting with the APEX2 tag itself. We also determined that HMGB1, which was detected in the APEX2 screen to bind to B1B2 greater than B1B2C, interacted in co-immunoprecipitation experiments with B1B2 better than B1B2C. Thus, the validation was successful in both directions.

Among the several RhoBTB1 binding partners derived from the proteomic screen, we chose SETD2 to further investigate. SETD2 is a histone lysine methyltransferase involved in epigenetic regulation, which may play a role in Huntington disease, Luscan-Lumish syndrome, Rabin–Pappas Syndrome, and Intellectual Developmental Disorder 70.^45–47^ We focused on SETD2 because it was reported to regulate coronary vascular development in the mouse heart and may contribute to pulmonary HTN.^48,49^ Indeed, we validated that SETD2 interacted with B1B2C and that its level of protein expression is markedly enhanced: (a) by an inhibitor of Cullin proteins (MLN4924) or the proteasome (MG132), (b) in HEK293 cells with an CRISPR-Cas9-induced mutation in CUL3, and (c) by inhibition of RhoBTB1. Because of its high molecular weight, we had difficulty determining if it is directly ubiquitinated by RhoBTB1-CUL3, but all other evidence supports that hypothesis. Future studies will explore the role of SETD2 in vascular function and BP regulation. Indeed, given the success of the approach undertaken in HEK293 cells, we are currently validating targets of RhoBTB1 interacting proteins identified in cultured smooth muscle cells, and plan to employ the technique in placental cells where the level of RhoBTB1 expression is among the highest across tissues.

## Supplementary Material

zqad034_Supplemental_Figures_and_Tables

## Data Availability

Mass spectrometry proteomics data have been deposited to the Proteome Xchange Consortium via the PRIDE partner repository with the dataset identifier PXD043245 and 10.6019/PXD043245. The data underlying this article will be shared on reasonable request to the corresponding author.
