# Supplementary material for: Structure and Function of RhoBTB1 Required for Substrate Specificity and Cullin-3 Ubiquitination
Source: Function (Oxf). 2023 Jul 3;4(5):zqad034. doi: 10.1093/function/zqad034 (PMC10413933; doi:10.1093/function/zqad034)
Supplement: zqad034_Supplemental_Figures_and_Tables [file zqad034_supplemental_figures_and_tables.zip › Supplemental Figures and Tables Revised.docx]

**Supplemental Figures**


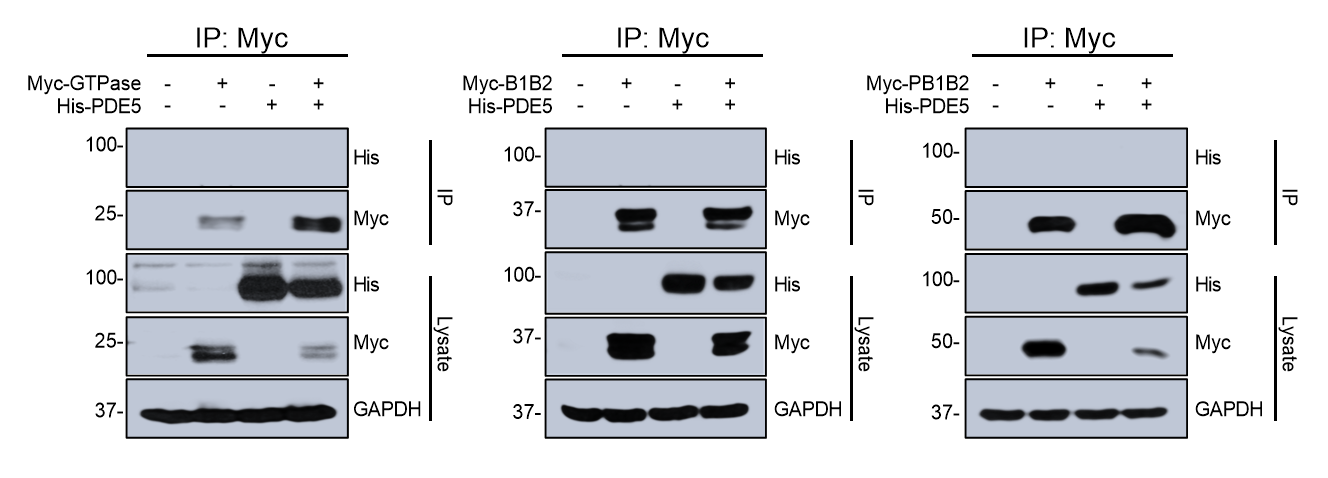


Figure S1. RhoBTB1 Domains Lacking C-Terminal Fail to Associate with PDE5.

HEK293 cells were transfected with either Myc-GTPase, Myc-B1B2 (lacking the C terminal, or Myc-PB1B2 (lacking the C terminal, and His-PDE5 for 16 hours and treated with 1 μM MLN4924 after 16 hours and 10 μM MG132 after 20 hours of transfection. Total transfection time was 24 hours. Whole-cell extracts were prepared and immunoprecipitated with the indicated antibodies, and immunoprecipitates were resolved by SDS-PAGE and immunoblotted with the indicated antibodies. IP and lysates are indicated. Molecular weight markers were transferred from the original blots. These data are representative of two independent experiments.


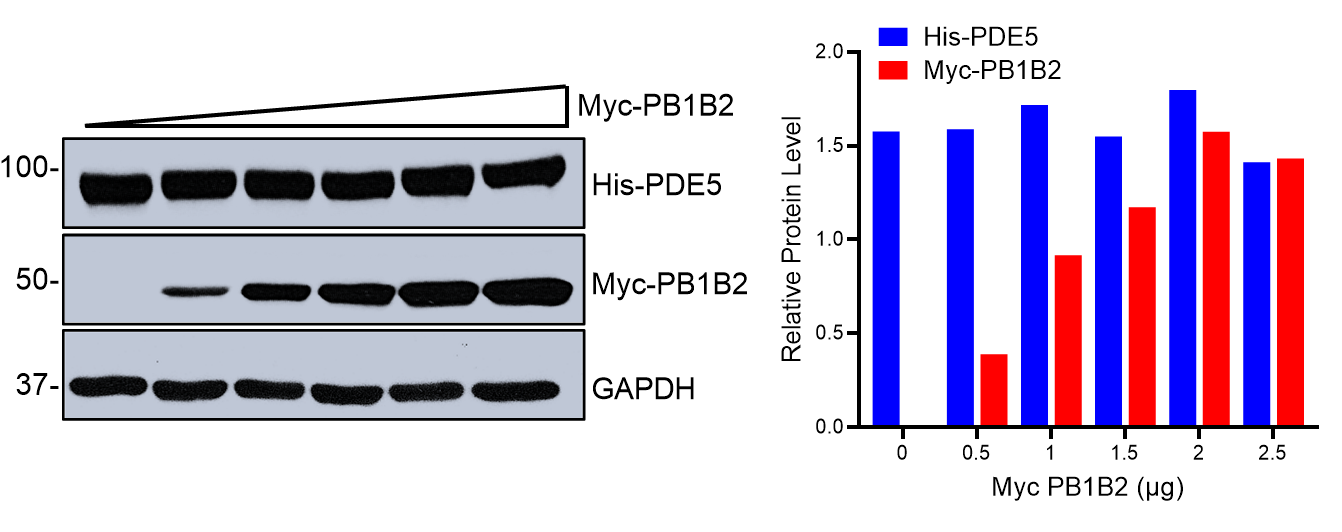


Figure S2. *PB1B2 Lacking the C-Terminal Domain is Insufficient to Regulate PDE5.*

HEK293 cells were transfected with the empty vector or Myc-PB1B2 and His-PDE5 for 16 hours, after which cells were treated with 100 μM cycloheximide for 8 hours. Cells were harvested, the whole-cell lysates were collected, and extracts were immunoblotted with the indicated antibodies. The relative levels of Myc-PB1B2 and PDE5 were quantified using ImageJ software. Data are average of duplicate experiments.


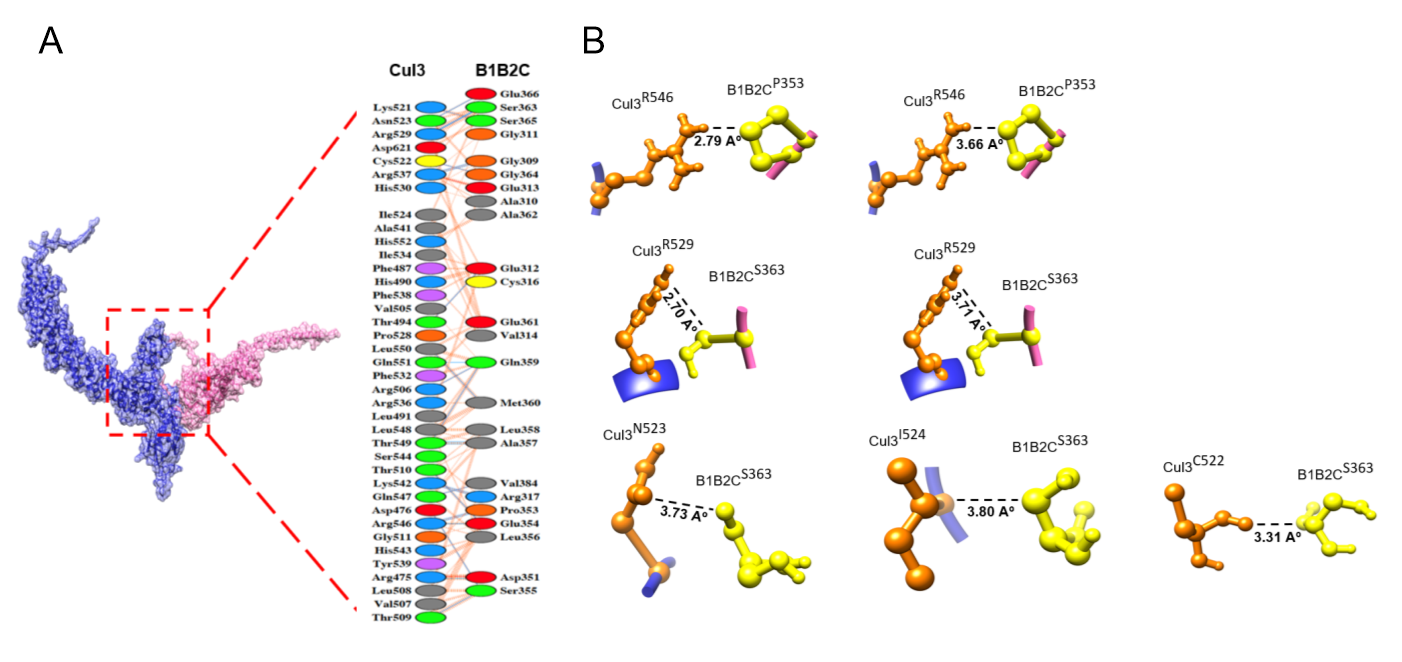


Figure S3. *Predicted Docking Complete for AlphaFold Structures of CUL3 and B1B2C*.

A) Docked complex comprising AlphaFold structures of CUL3 (blue) and B1B2C (magenta). Structures of CUL3 and RhoBTB1 were retrieved from AlphaFold and B1B2C was extracted from full-length AlphaFold structure of RhoBTB1. Molecular docking was performed by ClusPro and docking poses were filtered depending upon cluster with maximum numbers to visualize interface between CUL3 and B1B2C. B) The interacting amino acid residue pairs between CUL3 and B1B2C were predicted using PDBsum. Interacting amino acid pairs are shown and the interacting distances are labelled.


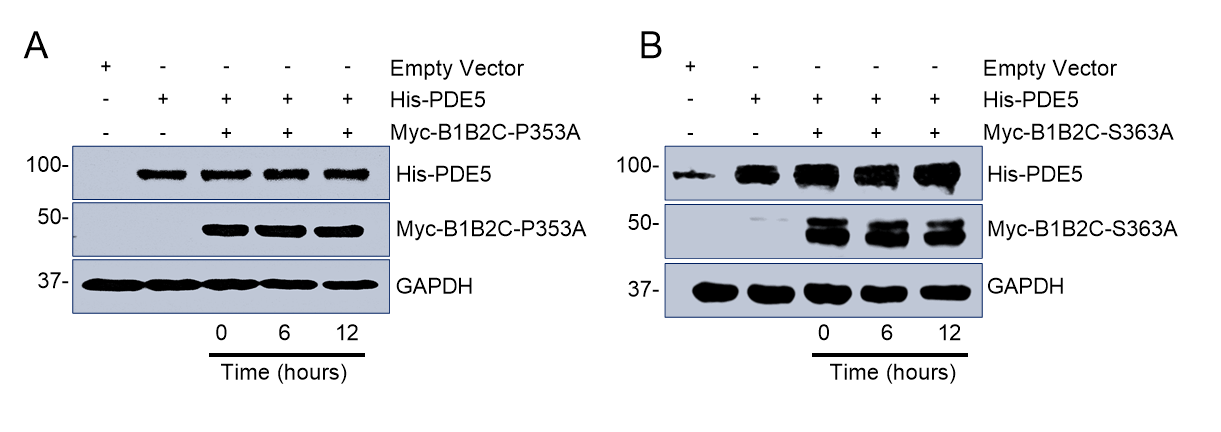


Figure S4. *RhoBTB1 Mutants Fail to Associate with CUL3 and to Degrade PDE5*.

A-B) HEK293 cells were transfected with the empty vector or Myc-B1B2C-P353A (A) or Myc-B1B2C-S363A (B) with His-PDE5 for 16 hours, after which the cells were treated with 100 μM cycloheximide for the indicated time. Cells were harvested, the whole-cell lysates were collected, and extracts were immunoblotted with the indicated antibodies. Molecular weight markers were transferred from the original blots. Data are representative of two independent experiments.


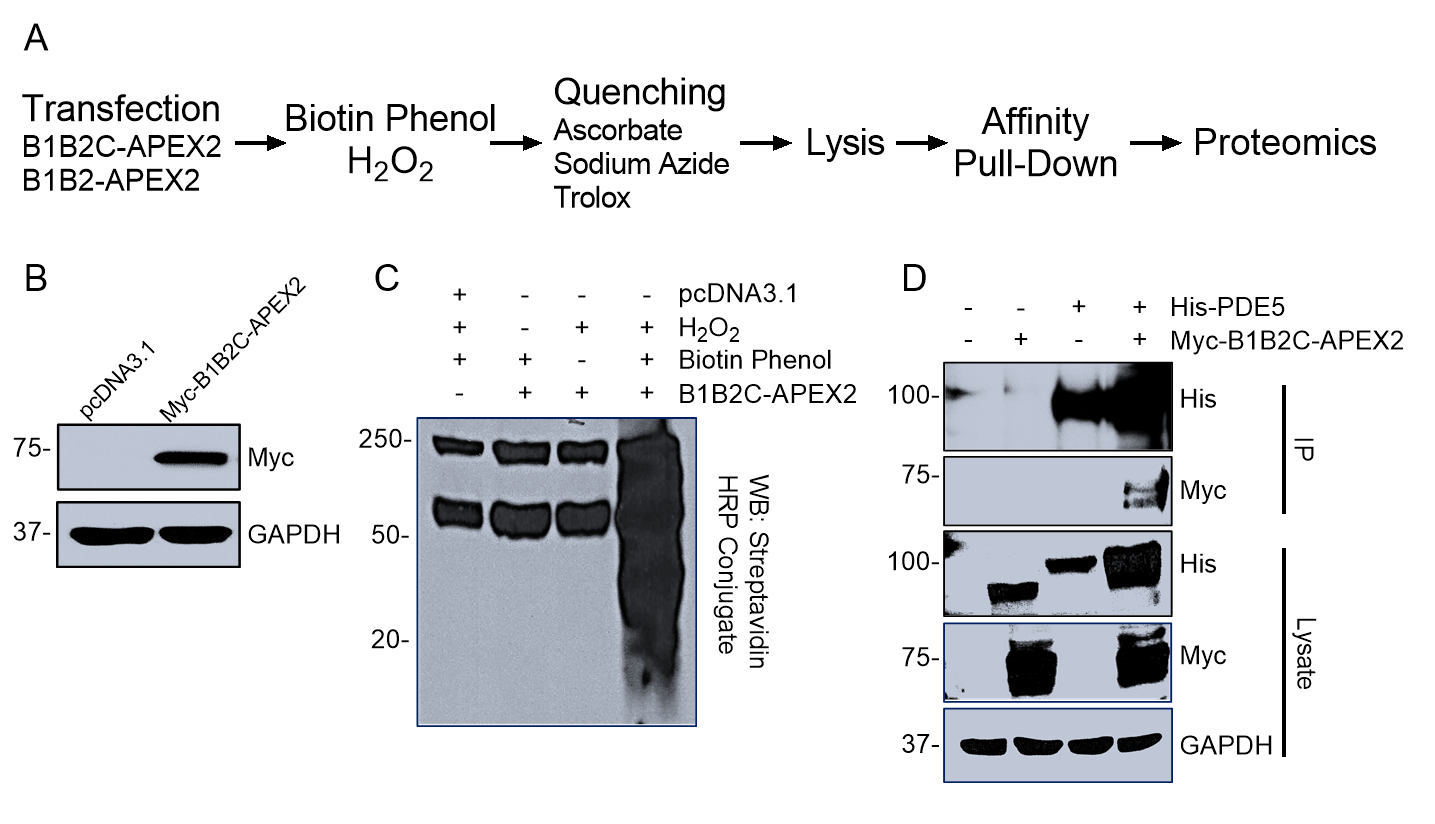


Figure S5. *Optimization of the APEX-2 Proximity Labeling Protocol*.

Schematic overview of APEX-2-mediated proximity labeling workflow in HEK293 cells. B) Expression of Myc-B1B2C-APEX2 fusion construct in HEK293 cells. C) Western blot analysis of biotinylated proteins by Myc-B1B2C-APEX2 in presence of H_2_O_2_ and biotin phenol. Blot was probed by streptavidin-HRP conjugate. D) Validation of interaction between PDE5 and Myc-B1B2C-APEX2 by Co-IP. Whole-cell extracts and immunoprecipitates were resolved by SDS-PAGE and blotted with the indicated antibodies. Molecular weight markers were transferred from the original blots.


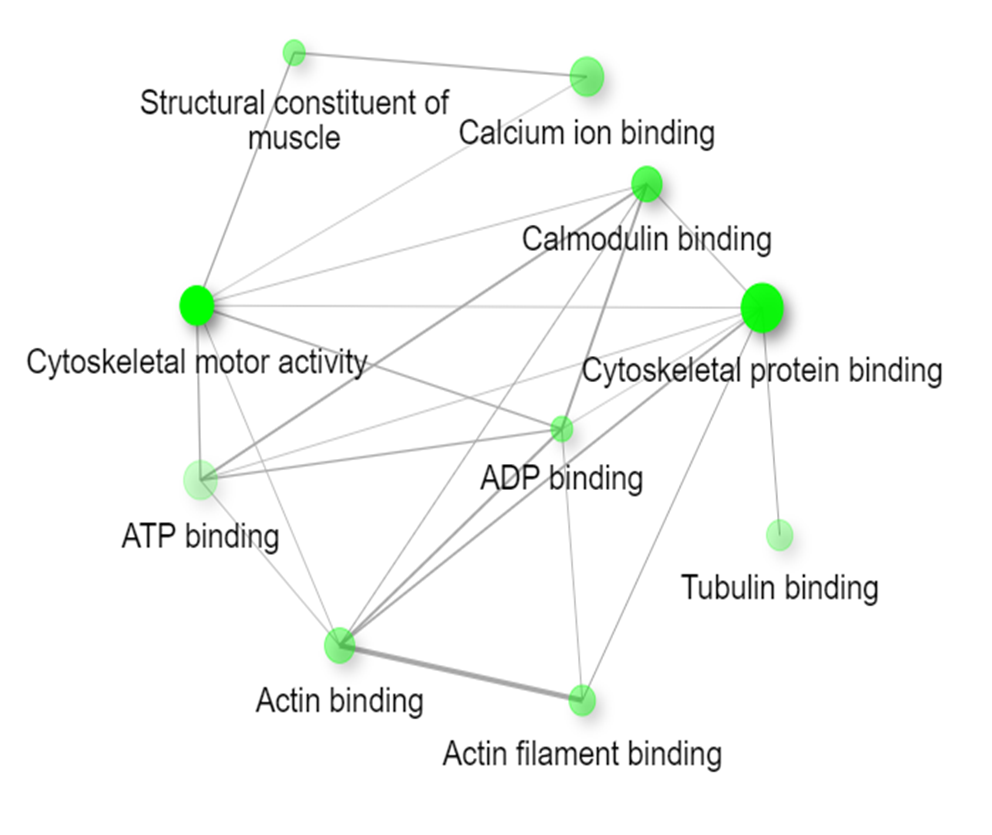


Figure S6. *Clustering of GO molecular functions was performed by ShinyGO*.

ShinyGO analysis of B1B2C interacting proteins revealed their involvement in calcium signaling, muscle contraction, nucleotide binding, and cytoskeletal remodeling. Darker nodes represent more significantly enriched gene sets and bigger nodes are larger gene sets while thicker edges represent more overlapped genes.

**Supplemental Tables**

Table S1. Primers used in site-directed mutagenesis

| Amino Acid | Forward Primer | Reverse Primer |
| --- | --- | --- |
| Pro353Ala | 5’ CAGGCCAGGACCTGGCTGAGAGTTTGGCTTTG 3’ | 5’ CAAAGCCAAACTCTCAGCCAGGTCCTGGCCTG 3’ |
| Ser363Ala | 5’ TGCAGATGGAAGCCGCGGGTTCCGAGGGACAC 3’ | 5’ GTGTCCCTCGGAACCCGCGGCTTCCATCTGCA 3’ |

Table S2: Primers used in real time RT PCR

| Gene | Forward Primer | Reverse Primer |
| --- | --- | --- |
| RhoBTB1_B1B2C | 5’ GAGCTGGCACAGTTTCACAA 3’ | 5’ TCTTCACACGCTGGTAGTGG 3’ |
| PDE5 | 5’ CCTACCTGGCATTCTGTGGT 3’ | 5’ GTGGCCGCTATCTTCTTCAG 3’ |
| GAPDH | 5’ GGTGAAGGTCGGAGTCAACG 3’ | 5’ AGGGATCTCGCTCCTGGAAG 3’ |

Table S3: siRNA used in RNA interference

| siRNA | Catalog No. | Sense | Anti-Sense |
| --- | --- | --- | --- |
| 1 | SASI_Hs01_00138546 | 5’-GAGUGUUUCUCUCAGGCUU (dT/dT)-3’ | 5’-AAGCCUGAGAGAAACACUC (dT/dT)-3’ |
| 2 | SASI_Hs01_00138547 | 5’-CUUUGCAUAUGGCAGGUCU (dT/dT)-3’ | 5’-AGACCUGCCAUAUGAAAAG (dT/dT)-3’ |
| 3 | SASI_Hs01_00138553 | 5’-GAACUUGGCUUACCAUACU(dT/dT)-3’ | 5’-AGUAUGGUAAGCCAAGUUC (dT/dT)-3’ |
| 4 | SIC001-10NMOL | Universal Negative control with Proprietary Sequence * | Universal Negative control with Proprietary Sequence * |

* Sequence not provided by supplier

Table S4. Chromatography and MS instrument acquisition settings

| Sample Volume | 20 µL | Isolation Window | 1.6 m/z |
| --- | --- | --- | --- |
| Stationary Phase | Thermo Acclaim PepMap C18  75µm × 50cm | MS2 AGC Target | 5e4 |
| LC Solvent A | 100% H2O,  0.1% formic acid | MS2 Maximum IT | 54 ms |
| LC Solvent B | 80% acetonitrile,  0.1% formic acid | Normalized Collision Energy | 30 |
| Gradient Ramp and Duration Flow Rate | 2.5-5% B in 1 minute  5-7% B in 4 minutes  7-28% B in 72 minutes  28-60%B in 10 minutes  60-99%B in 4 minutes  300 nL/min | Minimum Intensity Req. | 5e5 |
| Mass Spectrometer | Thermo Orbitrap Fusion Lumos | Dynamic Exclusion | 60.0 s |
| Spray Voltage | 2.1 kV | MS2 acquisition | Data dependent, 3 s cycle time, Centroid |
| In-Source CID | 0.0 eV | MS2 Fragmentation | HCD |
| MS1 scan range | 375-1500 m/z | MS2 Detection | Orbitrap |
| MS1 resolution | 120,000 @ 200 m/z | MS2 fixed first mass | 110 m/z |
| MS1 AGC Target | 4e5 | MS2 resolution | 30,000 @ 200 m/z |
| MS1 Maximum IT | 50 ms | Advanced Precursor Determination | on |

Table S5. Mass spectrometry data processing parameters

| Platform | ProteomeDiscoverer 2.4 | Quantitation | Precursor Ions Quantifier, Peak Area |
| --- | --- | --- | --- |
| Search Algorithms | SequestHT | Normalization | Total Peptide Amount |
| Validation | Percolator | Scaling | Validation |
| Database | Uniprot Rattus norvegicus with isoforms, 2022-04-30, MaxQuant Contaminants  Recombinant sequence | Ratio Calculation | Summed Abundance Based |
| Digest | Trypsin (semi)  2 Missed Cleavages Allowed | Hypothesis Test | ANOVA (Individual Proteins) |
| Precursor mass tolerance | 10 ppm | Target FDR (Strict) for PSMs: | 0.01 |
| Fragment mass tolerance | 0.02 Da | Target FDR (Relaxed) for PSMs: | 0.05 |
| Static Modifications | Carbamidomethyl (C) | Target FDR (Strict) for Peptides: | 0.01 |
| Dynamic Modifications | Oxidation (M), Acetylation (protein N-terminus) | Target FDR (Relaxed) for Peptides: | 0.05 |
